# Chromatin CKAP2, a New Proliferation Marker, as Independent Prognostic Indicator in Breast Cancer

**DOI:** 10.1371/journal.pone.0098160

**Published:** 2014-06-02

**Authors:** Han-Seong Kim, Jae-Soo Koh, Yong-Bock Choi, Jungsil Ro, Hyun-Kyoung Kim, Mi-Kyung Kim, Byung-Ho Nam, Kyung-Tae Kim, Vishal Chandra, Hye-Sil Seol, Woo-Chul Noh, Eun-Kyu Kim, Joobae Park, Chang-Dae Bae, Kyeong-Man Hong

**Affiliations:** 1 Department of Pathology, Inje University Ilsan Paik Hospital, Ilsanseo-gu, Goyang, Korea; 2 Department of Pathology, Korea Cancer Center Hospital, Nowon-gu, Seoul, Korea; 3 Research Institute, National Cancer Center, Ilsandong-gu, Goyang, Korea; 4 Department of Surgery, Breast Cancer Center, Korea Cancer Center Hospital, Nowon-gu, Seoul, Korea; 5 Department of Molecular Cell Biology, Sungkyunkwan University School of Medicine, and Samsung Biomedical Research Institute, Suwon, Korea; Health Canada and University of Ottawa, Canada

## Abstract

**Background:**

The level of proliferation activity is a strong prognostic or predictive indicator in breast cancer, but its optimal measurement is still in debate, necessitating new proliferation markers. In the present study, the prognostic significance of the CKAP2-positive cell count (CPCC), a new proliferation marker, was evaluated, and the results were compared with those for the mitotic activity index (MAI).

**Methods:**

This study included 375 early-stage breast cancer samples collected from two institutions between 2000 and 2006. Immunohistochemical staining was performed using a CKAP2 monoclonal antibody. Cox proportional hazard regression models were fitted to determine the association between the CPCC and relapse-free survival (RFS) amongst three groups formed on the basis of the CPCC or MAI value: groups 2 and 3 showing the middle and highest values, respectively, and group 1 the lowest.

**Results:**

After adjustment for age, T stage, N stage, HER2 status, estrogen receptor status, progesterone receptor status, institution, and year of surgical resection, the CPCC was associated with a significantly worse RFS {hazard ratio [HR]  = 4.10 (95% CI: 1.64–10.29) for group 2; HR  = 4.35 (95% CI: 2.04–10.35) for group 3}. Moreover, its prognostic significance was similar to or higher than that based on the MAI {HR  = 2.05 (95% CI: 0.94–4.65) for group 2; HR  = 2.35 (95% CI: 1.09–5.10) for group 3}. In subgroup analyses, the CPCC showed a prognostic significance in the luminal A and triple-negative subgroups, but not in the HER2-positive subgroup.

**Conclusions:**

Chromatin CKAP2 is an independent prognostic marker for RFS in early-stage breast cancer, and could potentially replace the MAI in clinical evaluation of proliferation activity. Additionally, our study results suggest that the prognostic significance of proliferation activity differs among the various subgroups of breast cancer.

## Introduction

Proliferation activity has been recognized as one of the most reliable breast cancer prognosticators [1], [2], [3]. Moreover, it has been identified as a reliable predictive marker for anti-cancer therapy, with higher proliferation activity correlating with stronger response to chemotherapy [4], [5]. So, in addition to classical mitotic counting, a number of markers, including Ki-67, cyclin D, cyclin E, p27, p21, thymidine kinase, topoisomerase IIα, and phosphohistone H3, have been used to measure proliferation activity [6], [7]. However, debate continues over which proliferation marker is the most reliable for clinical application. For example, whereas the mitotic activity index (MAI) has been the most reliable breast cancer prognosticator [1], [2], the clinical application data on Ki-67 has been inconclusive [8], [9]. On the contrary, as a predictive marker in breast cancer, Ki-67 has been the most widely evaluated, showing its clinical applicability, especially in triple-negative (TN) breast cancer [5], [10], [11]. Clearly, further prognostic and predictive evaluations of the currently available markers are necessary, and development of new proliferation markers, in turn, could facilitate the clinical application of proliferation activity to breast cancer.

Cytoskeleton-associated protein 2 (CKAP2) [or tumor-associated microtubule-associated protein/cytoskeleton-associated protein 2 (TMAP/CKAP2)] is a microtubule-associated protein that plays key roles in the regulation of microtubule assembly and disassembly, not to mention kinetochore and microtubule attachment during mitosis and cytokinesis [12], [13], [14]. We previously demonstrated both the localization of CKAP2 in the condensed chromatin of mitotic cells and the close correlation of chromatin CKAP2-positive cell count (CPCC) with the mitotic figure count [14], [15], indicating that chromatin CKAP2 is another proliferation marker with specificity in the mitotic phase. However, its prognostic significance has not been evaluated for any cancer. Therefore, in the present study, the prognostic significance of chromatin CKAP2 was evaluated in 375 early-stage breast cancer cases, from two independent institutions, as based on the CPCC in CKAP2 immunohistochemistry.

## Materials and Methods

### Breast cancer tissues

Formalin-fixed, paraffin-embedded breast cancer tissues representing a total of 375 invasive breast cancer cases, 266 from the Korea Cancer Center Hospital (KCCH; 2005–2006) and 109 from Ilsan Inje Paik Hospital (IIPH; 2000–2003), were studied. Access to and usage of clinical information and the relevant archival tissues were approved by the Institutional Review Boards of the National Cancer Center, the KCCH, and IIPH, which waived the need for informed consent. The relevant clinical characteristics are listed in Table 1. The estrogen receptor (ER) and progesterone receptor (PR) positivity statuses, as based on hospital records, were determined by Allred score, according to which, intermediate or strong hormone receptor cases are counted as positive. The human epidermal growth receptor 2 (HER2) status also based on hospital records, was determined by IHC staining: 3+ is counted as positive; 0 or 1+, negative. In cases with 2+ in IHC staining, FISH is performed, counting copy number 4 or more as positive.

**Table 1:** Clinicopathological characteristics of breast cancer patients.

Clinical Variables	Total (%)	KCCH (%)	IIPH (%)
Number	375	266	109
Sex			
Male	1 (0.3)	0	1 (0.9)
Female	374 (99.7)	266	108 (99.1)
Median age at diagnosis (year, quartile range)	48 (42–57.5)	49 (44–58)	45 (39–57)
Median follow up (month, quartile range)	51.3 (30.9–61.5)	48.5(30.4–56.1)	77.6(36.1–97.7)
Histology			
Invasive ductal carcinoma	363 (96.8)	255 (95.9)	108 (99.1)
Not otherwise specified (NOS)	337 (89.9)	244 (91.7)	93 (85.3)
Mucinous	11 (2.9)	4 (1.5)	7 (6.4)
Papillary	2 (0.5)	2 (0.8)	0
Metaplastic	3 (0.8)	3 (1.1)	0
Apocrine	3 (0.8)	2 (0.8)	1 (0.9)
Others	7 (1.9)	0	7 (6.4)
Invasive lobular carcinoma	12 (3.2)	11 (4.1)	1 (0.9)
T stage			
1	86 (22.9)	38 (14.3)	48 (44.0)
2	277 (73.9)	228 (85.7)	49 (45.0)
3	12 (3.2)	0	12 (11.0)
N stage			
0	183 (48.8)	129 (48.5)	54 (49.5)
1	101 (26.9)	77 (28.9)	24 (22.0)
2	64 (17.1)	42 (15.8)	22 (20.2)
3	24 (6.4)	18 (6.8)	6 (5.5)
Unknown	3 (0.8)	0	3 (2.8)
ER			
Negative	184 (49.1)	135 (50.8)	49 (45.0)
Positive	186 (49.6)	128 (48.1)	58 (53.2)
Unknown	5 (1.3)	3 (1.1)	2 (1.8)
PR			
Negative	167 (44.5)	116 (43.6)	51 (46.8)
Positive	203 (54.1)	147 (55.3)	56 (51.4)
Unknown	5 (1.3)	3 (1.1)	2 (1.8)
HER2/*neu*			
Negative	251 (66.9)	177 (66.5)	74 (67.9)
Positive	90 (24.0)	60 (22.6)	30 (27.5)
Unknown	34 (9.1)	29 (10.9)	5 (4.6)
Recurrence			
Yes	69 (18.4)	43 (16.2)	26 (23.9)
No	293 (78.1)	218 (82.0)	75 (68.8)
Unknown	13 (3.5)	5 (1.9)	8 (7.3)
Subgroup			
Luminal A*	164 (43.7)	114 (42.9)	50 (45.9)
HER2-Positive**	90 (24.0)	60 (22.6)	30 (27.5)
Triple-Negative***	87 (23.2)	63 (23.7)	24 (22.0)
Unknown	34 (9.1)	29 (10.9)	5 (4.6)
CPCC (quartile range)	23 (11–44)	26.5 (14–47.5)	14 (6–37)
MAI (quartile range)	10 (3–20)	11 (4–21.8)	6 (2–16)

^*^Luminal A subgroup: cases with hormone receptor (HR)-positive and HER2-negative status.

^**^HER2-positive subgroup: cases with HER2-positive status with or without HR positivity.

^***^Triple-negative subgroup (TN): HR-negative and HER2-negative status.

IIPH  =  Ilsan Inje Paik Hospital; KCCH  =  Korean Cancer Center Hospital; ER  =  estrogen receptor; PR  =  progesterone receptor; HR  =  hormone receptor; HER2  =  human epidermal growth factor receptor 2; CPCC  =  chromatin CKAP2-positive cell count; MAI  =  mitotic activity index.

### Immunohistochemistry with monoclonal human CKAP2 antibody

Immunohistochemical staining (IHC) was performed using the Ultravision LP Detection System (Thermo Fisher Scientific Inc., Fremont, CA) as previously described using the same CKAP2 antibody [15]. Briefly, following deparaffinization of formalin-fixed, paraffin-embedded breast cancer tissues, antigen was retrieved in 10 mM citrate buffer, pH 6.0, containing 0.05% Tween 20. After ethanol fixation, the tissues were sequentially treated with 3% hydrogen peroxide and Ultra V block solution. After 1 h room-temperature incubation with anti-CKAP2 antibody, the slides were washed in Tris-buffered saline including Tween 20 (TBST), incubated with primary antibody enhancer for 10 min, and exposed to horseradish peroxidase-conjugated secondary antibody for 15 min. After re-washing in TBST, the tissue slides were incubated with diaminobenzidine chromogen (Scytek Laboratories Inc, Logan, UT) and counter-stained with Mayer’s hematoxylin (Dako Cytomation, Glostrup, Denmark).

### Evaluation of chromatin CKAP2-positive cell count and MAI

The CPCC was determined by counting the total number of CKAP2-positive cells per 10 consecutive high-power (400×) fields in the area with the highest number of chromatin CKAP2-positive cells. In the evaluation of the CKAP2-positive cells, strongly- to moderately-stained chromatin-positive cells were included. The inter-observer CPCC correlation was evaluated by two independent observers for 100 cases among the KCCH tissues.

The MAI was determined as previously reported [16]. Briefly, it was estimated on H&E-stained slides by summing the number of mitotic cells identified under 10 consecutive 400× power fields. The inter-observer MAI correlation was evaluated by two independent observers for the same 100 cases as just noted for the CPCC.

### Statistical analysis

A two-sided Spearman’s rank correlation test or Wilcoxon rank sum test was applied to correlation analyses of the CPCC or MAI with clinicopathological data, considering *P* values less than 0.05 as statistically significant. Both the inter-observer correlation of the CPCC or MAI and the correlation between them were tested with the two-sided Spearman correlation test.

To estimate the prognostic significance of chromatin CKAP2, the total number of cases was equally divided into three groups based on the CPCC values: group 1, ≤14 (N = 126); group 2, 15–36 (N = 114); group 3, ≥37 (N = 122). The same classification was performed based on the MAI values: group 1, ≤5 (N = 124); group 2, 6–16 (N = 116); group 3, ≥17 (N = 122).

In order to investigate the prognostic significance of the CPCC or MAI in the breast cancer subgroups, all of the cases were classified, based on the ER, PR, and HER2 statuses, into three subgroups: 1) the luminal A subgroup showing hormone receptor (HR)-positive and HER2-negative receptor status; 2) the HER2-positive subgroup; 3) the triple-negative (TN) subgroup being negative for all three receptors. Whereas the luminal B cases showing both HR-positive and HER2-positive status were included in the HER2-positive subgroup, the luminal B cases showing HR-positive, HER2-negative, and high Ki-67 level were included in luminal A subgroup, in the present study.

Relapse-free survival (RFS) was defined as the time from radical surgical resection to diagnosis of relapse, or the last date of follow-up. RFS estimates were calculated by the Kaplan-Meier method, and differences were assessed by log-rank test. Additionally, Kaplan-Meier survival graphs stratified by breast cancer subgroup were generated. Multivariate analyses were carried out using Cox’s proportional hazard regression model (hazard ratios with their 95% CIs) after adjustment for age (10-year age groups), T stage, N stage, HER2 status, estrogen receptor status, progesterone receptor status, institution, and year of surgical resection. Linear trends were calculated using the median value for each exposure variable as a continuous variable. Multivariate analyses were performed on the various breast cancer subgroups after adjustment for age, T stage, N stage, institution, and year of surgical resection. The statistical analyses were performed with GraphPad Prism version 5 (GraphPad Software, Inc., San Diego, CA) or SPSS version 18 (SPSS, Inc., Chicago, IL).

## Results

### Chromatin localizations of CKAP2 in breast cancer tissues

**Figure 1:**
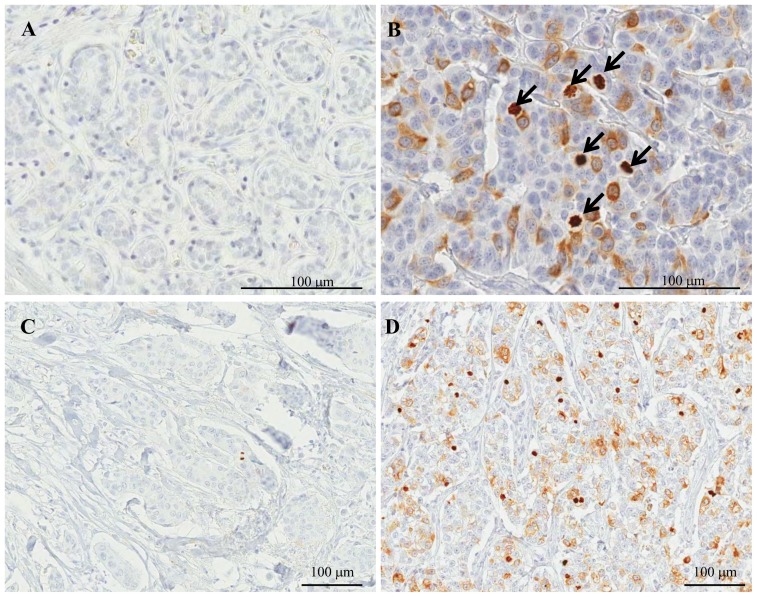
CKAP2 immunohistochemical staining in breast cancer tissues. CKAP2-positive cells are rare in normal breast ductal cells (A), but present variably in breast cancer tissues (B). The chromatin CKAP2-positive cell numbers were variable: low as in (C), or high as in (D). One hundred µl rulers are shown. The arrows indicate chromatin CKAP2 staining.

Immunohistochemical staining in normal breast tissues adjacent to cancer cells revealed rare CKAP2 staining (Fig. 1A), but in breast cancer tissues, CKAP2 was localized in the condensed chromatin of the mitotic cells (Fig. 1B, arrows). Only moderately- to strongly-stained chromatin CKAP2-positive cells were included in the CPCC evaluation. The numbers of chromatin-stained CKAP2-positive cells varied according to the breast cancer cases (Figs. 1C-D).

**Figure 2:**
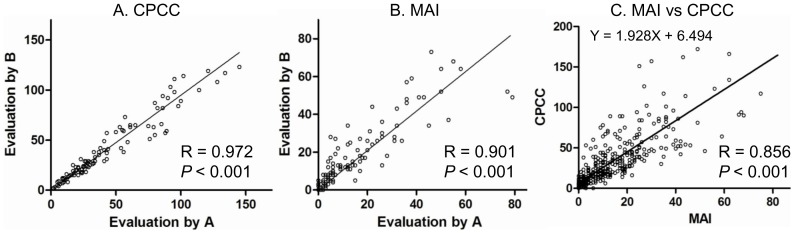
Inter-observer correlations of CPCC or MAI and correlation between CPCC and MAI. A. Inter-observer correlation of CPCC among 100 cases. B. Inter-observer correlation of MAI among 100 cases. C. Correlation between CPCC and MAI in total 375 cases. Two data points are outside the axis limits in C. The slope and Y intercept are shown. The correlations were calculated by two-sided Spearman test. CPCC  =  chromatin CKAP2-positive cell count; MAI  =  mitotic activity index.

The inter-observer correlations for both the CPCC (R = 0.972, *P*<0.001) and the MAI (R = 0.901, *P*<0.001) were high, as shown in Figs. 2A and 2B, respectively, suggesting that the evaluations were quite reproducible. The correlation between the CPCC and the MAI was also high (R = 0.856, *P*<0.001, Fig. 2C), suggesting the CPCC’s utility as an index for proliferation activity in cancer. The value of the CPCC was 1.928 times higher than that of the MAI with the Y-intercept of 6.494.

### Clinical characteristics of study population

The clinical characteristics of the breast cancer patients are listed in Table 1. As already noted, a total of 375 invasive breast cancer cases, 266 from the KCCH and 109 from IIPH, were analyzed. Whereas cases for the years 2000–2003 were randomly selected at IIPH, T2 cases (85.7% of the total) were preferentially selected at the KCCH. The T stage was higher at the KCCH, due to the preferential T2 selection; the CPCC and MAI levels were higher at the KCCH as well, a fact which might also be related to preferential T2 selection.

### Correlations between CPCC and clinicopathological factors

The CPCC distribution was 0–296 (quartile range: 11–44), with the median value of 23 (Fig. 2A and Table 1). An analysis of the CPCC’s association with the clinicopathological factors revealed a significant correlation with T stage (ρ = 0.219, *P*<0.001 by Spearman correlation test), estrogen receptor status (Z = −7.25, *P*<0.001 by Wilcoxon rank sum test), and progesterone receptor status (Z = −6.57, *P*<0.001), but not with N stage (ρ = 0.08, *P* = 0.113) or HER2 status (Z = −1.57, *P* = 0.116). Similarly, MAI showed significant correlations with clinicopathological parameters such as T stage (ρ = 0.166, *P*<0.001), estrogen receptor status (Z = −6.394, *P*<0.001), and progesterone receptor status (Z = −3.891, *P*<0.001), but not with N stage (ρ = 0.019, *P* = 0.713) or HER2 status (Z = −1.47, *P* = 0.143).

### Prognostic significance of chromatin CKAP2 expression

**Figure 3:**
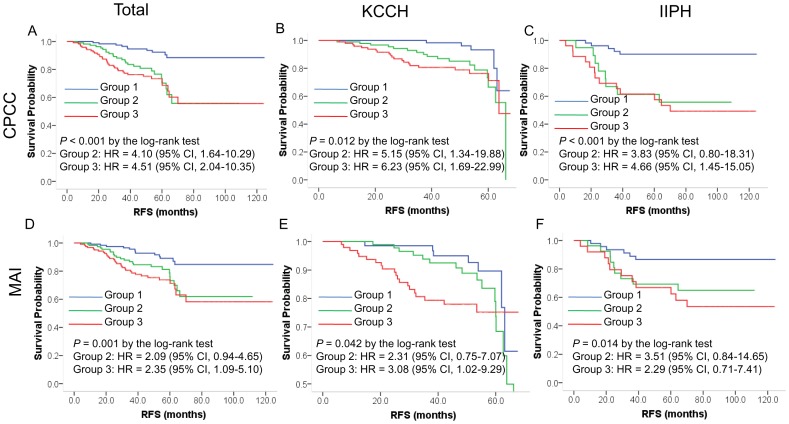
Correlations of CPCC and MAI with RFS. Shown are a Kaplan-Meier CPCC plot for total, KCCH, or IIPH cases (A-C) and an MAI plot for total, KCCH, or IIPH cases (D-F). The *P* values were determined by log-rank test, and the hazard ratios (HRs) and their 95% confidence interval (CI) by the Cox proportional hazard regression model according to the co-variables of age, T stage, N stage, HER2 status, estrogen receptor status, progesterone receptor status, institution, and year of surgical resection. The X-axis is RFS in months, and the Y-axis, RFS probability. CPCC  =  chromatin CKAP2-positive cell count; MAI  =  mitotic activity index.

As for RFS, the CPCC showed a significant correlation by log-rank test in the total cases (Fig 3A, *P*<0.001), the KCCH cases (Fig. 3B, *P* = 0.012), and the IIPH cases (Fig. 3C, *P*<0.001). Likewise, the MAI showed a significant correlation with RFS in the total cases (Fig 3D, *P* = 0.001), the KCCH cases (Fig. 3E, *P* = 0.042), and the IIPH cases (Fig. 3F, *P* = 0.014).

In univariate analyses, factors including age, T stage, N stage, and HER2 status were significantly correlated with RFS (Table 2). The CPCC and MAI showed a significant correlation with RFS across the two institutions, but the significance was higher with the CPCC than with the MAI in terms of hazard ratios (HRs) and *P* values (Table 2).

**Table 2:** Univariate analysis of clinicopathological factors for correlation with RFS.

Variable	Group	Range of variable	Total		KCCH		IIP	
			HR (95% CI)	*P*	HR (95% CI)	*P*	HR (95% CI)	*P*
Age	Group 1	−39						
	Group 2	40–49	0.46 (0.23–0.91)	.026	0.30 (0.12–0.74)	.009	0.65 (0.23–1.88)	.428
	Group 3	50–59	0.78 (0.40–1.52)	.463	0.64 (0.29–1.43)	.275	0.49 (0.10–2.30)	.366
	Group 4	60–69	1.14 (0.56–2.33)	.725	0.77 (0.30–1.98)	.591	1.60 (0.52–4.91)	.409
	Group 5	70-	1.52 (0.60–3.85)	.375	0.35 (0.04–2.71)	.312	2.85 (0.93–8.75)	.068
T stage	T1							
	T2		2.19 (1.10–4.34)	.025	2.41 (0.85–6.83)	.098	2.73 (0.98–7.57)	.054
	T3		5.99 (2.28–15.76)	<.001			6.34 (2.01–20.07)	.002
N stage	No							
	N1		2.64 (1.38–5.06)	<.001	2.18 (0.99–4.81)	.053	3.01 (1.05–10.42)	.041
	N2		5.11 (2.68–9.75)	<.001	4.02 (1.77–9.13)	.001	6.85 (2.33–20.13)	<.001
	N3		6.56 (2.98–14.48)	<.001	4.70 (1.71–12.94)	.003	9.38 (2.52–34.98)	<.001
Histology	NOS invasive ductal carcinoma							
	Other invasive ductal carcinoma		0.53 (0.17–1.68)	.280	2.07 (0.49–8.68)	.319	0.21 (0.03–1.55)	.125
	Invasive lobular carcinoma		1.31 (0.41–4.16)	.651	1.57 (0.48–5.11)	.457	-	-
HER2	Negative							
	Positive		1.73 (1.03–2.89)	.037	1.34 (0.67–2.68)	.402	2.46 (1.13–5.37)	.023
ER	Negative							
	Positive		0.731 (0.44–1.18)	.198	0.89 (0.482–1.63)	.705	0.52 (0.24–1.12)	.096
PR	Negative							
	Positive		0.70 (0.41–1.08)	.098	0.71 (0.38–1.30)	.263	0.66 (0.31–1.45)	.303
CPCC	Group 1	0–14						
	Group 2	15–36	3.88 (1.86–8.13)	<.001	3.20 (1.18–8.68)	.022	5.35 (1.75–16.78)	.003
	Group 3	37-	4.82 (2.37–9.79)	<.001	3.97 (1.50–10.56)	.006	6.50 (2.32–18.23)	<.001
MAI	Group 1	0–5						
	Group 2	6–16	2.41 (1.22–4.73)	.011	2.14 (0.87–5.25)	.097	2.88 (1.03–8.10)	.045
	Group 3	17-	3.23 (1.69–6.17)	<.001	2.89 (1.23–6.81)	.015	3.97 (1.47–10.75)	.007

* KCCH  =  Korean Cancer Center Hospital; IIPH  =  Ilsan Inje Paik Hospital; CI  =  confidence interval; HER2  =  human epidermal growth factor receptor 2; ER  =  estrogen receptor; PR, progesterone receptor; NOS, not otherwise specified.

–: not analyzed due to limited number of cases.

In multivariate analyses using the Cox proportional hazard regression model with co-variables including age, T stage, N stage, HER2 status, ER status, PR status, institution, and year of surgical resection, the CPCC showed significant correlations with worse RFS in the total cases for groups 2 and 3, the KCCH cases for groups 2 and 3, and the IIPH cases for group 3 (Table 3). In multivariate analyses with the same co-variables as for the CPCC, the MAI showed significant correlations with worse RFS in the total cases and KCCH cases for group 3, but not in the IIPH cases. The significance, once again, was higher with the CPCC than with the MAI in terms of HRs and *P* values (Table 3)

**Table 3:** Multivariate analysis of CPCC or MAI for correlation with RFS using Cox proportional hazard regression model at two institutions.

Parameter	Group	Range of variable	Total		KCCH		IIP	
			HR (95% CI)	*P*	HR (95% CI)	*P*	HR (95% CI)	*P*
CPCC	Group 1	0–14	1		1		1	
	Group 2	15–36	4.10 (1.64–10.29)	.003	5.15 (1.34–19. 88)	.017	3.83 (0.80–18.31)	.093
	Group 3	37-	4.51 (2.04–10.35)	<.001	6.23 (1.69–22.99)	.006	4.66 (1.45–15.05)	.010
				.002†		.023†		.034†
MAI	Group 1	0–5	1		1		1	
	Group 2	6–16	2.09 (0.94–4.65)	.071	2.31 (0.75–7.07)	.144	3.51 (0.84–14.65)	.085
	Group 3	17-	2.35 (1.09–5.10)	.030	3.08 (1.02–9.29)	.046	2.29 (0.71–7.41)	.167
				.088†		.138†		.208†

The co-variables of age at diagnosis (groups 1–5), T stage (T1, T2 or T3), N stage (N0, N1, N2 or N3), HER2 status (negative or positive), ER status (negative or positive), PR status (negative or positive), institution (KCCH or IIPH), and year of surgical resection (2000, 2001, 2002, 2003, 2005, or 2006) were used in the multivariate analysis, treating each co-variable as a categorical variable.

† *P* for linear trend.

KCCH  =  Korean Cancer Center Hospital; IIPH  =  Ilsan Inje Paik Hospital; CI  =  confidence interval.

### Prognostic significance in breast cancer subgroups

Among the breast cancer subgroups, RFS differed significantly (*P* = 0.014, by log-rank test): the best was the luminal A subgroup, and the worst, the HER2-positive subgroup.

**Figure 4:**
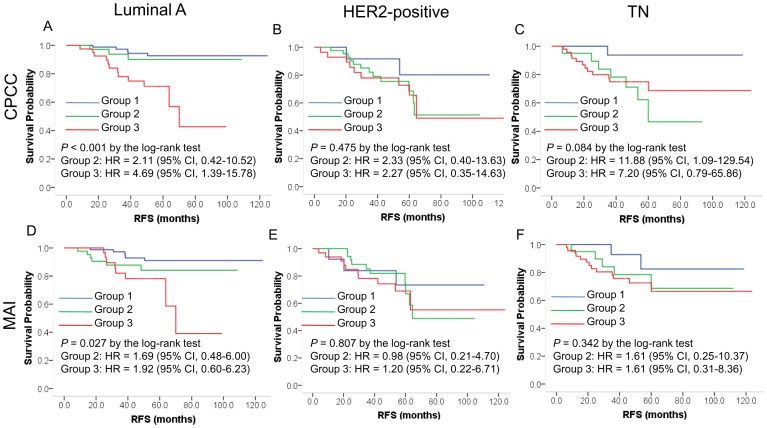
Prognostic significance of proliferation activity in breast cancer subgroups. Shown are Kaplan-Meier CPCC plots for the luminal A (A), HER2-positive (B), and TN (C) subgroup cases, as well as MAI plots for the luminal A (D), HER2-positive (E), and TN (F) subgroup cases. The *P* values were determined by log-rank test, and the hazard ratios (HRs) and their 95% confidence interval (CI) by the Cox proportional hazard regression model according to the co-variables of age, T stage, N stage, institution, and year of surgical resection. The X-axis is RFS in months, and the Y-axis, RFS probability. CPCC  =  chromatin CKAP2-positive cell count; MAI  =  mitotic activity index.

The CPCC and MAI showed significant correlations with RFS in the luminal A subgroup, and the CPCC showed a marginal correlation in the TN subgroup, by log-rank test (Fig. 4). In the HER2-positive subgroup, however, neither the CPCC nor the MAI showed any significant correlations with RFS (Fig. 4), suggesting that proliferation activity has different prognostic roles in the various breast cancer subgroups. In a Kaplan-Meier plot, CPCC group 2 showed different patterns in the luminal A and TN subgroups: in the luminal A subgroup, the RFS curve for group 2 was similar to that for group 1, but in the TN subgroup, it was similar to that for group 3 (Fig. 4), suggesting that the CPCC-based prognostic significance in group 2 differs between the luminal A and TN subgroups.

In the multivariate analysis using age, T stage, N stage, institution, and year of surgical resection as the co-variables, the CPCC showed a significant correlation with RFS in the luminal A (HR = 4.69 with 95% CI = 1.39–15.78, for group 3) and TN (HR = 11.88 with 95% CI = 1.09–129.54, for group 2) subgroups (Table 4). CPCC subgroup 3 showed also a marginal correlation with worse RFS in the TN subgroup (Table 4). These data again suggest that the prognostic significance of proliferation activity based on CPCC differ among the various subgroups. However, the MAI did not show any significant correlation with RFS among the various breast cancer subgroups (Table 4).

**Table 4:** Multivariate analysis of CPCC or MAI for correlation with RFS in breast cancer subgroups by Cox proportional hazard regression model.

Parameter	Group	Range of variable	Luminal A*		HER2-positive**		TN***	
			HR (95% CI)	*P*	HR (95% CI)	*P*	HR (95% CI)	*P*
CPCC	Group 1	0–14	1		1		1	
	Group 2	15–36	2.11 (0.42–10.52)	.362	2.33 (0.40–13.63)	.348	11.88 (1.09–129.54)	.042
	Group 3	37-	4.69 (1.39–15.78)	.013	2.27 (0.35–14.63)	.389	7.20 (0.79–65.86)	.080
				.038†		.637†		.127†
MAI	Group 1	0–5	1		1		1	
	Group 2	6–16	1.69 (0.48–6.00)	.414	0.98 (0.21–4.70)	.983	1.61 (0.25–10.37)	.619
	Group 3	17-	1.92 (0.60–6.23)	.275	1.20 (0.22–6.71)	.833	1.61 (0.31–8.36)	.574
				.528†		.932†		.850†

The co-variables of age at diagnosis (groups 1–5), T stage (T1, T2 or T3), N stage (N0, N1, N2 or N3), institution (KCCH or IIPH), and year of surgical resection (2000, 2001, 2002, 2003, 2005 or 2006) were used in the multivariate analysis, treating each co-variable as a categorical variable.

*Luminal A subgroup: cases with hormone receptor (HR)-positive and HER2-negative status.

**HER2-positive subgroup: cases with HER2-positive status with or without HR positivity.

***Triple-negative subgroup (TN): HR-negative and HER2-negative status.

† *P* for linear trend.

## Discussion

The present study by means of an immunohistochemical evaluation of chromatin CKAP2-positive cell counts, showed that breast cancer with higher CPCC values was significantly correlated with worse RFS in the multivariate analyses across two independent institutions. Moreover, the prognostic significance of the CPCC was higher than that of the MAI in terms of HRs and *P* values. Thus, a proliferation marker, chromatin CKAP2, might be a new useful and alternative prognostic tool to the MAI in breast cancer. Of note, however, the CPCC showed prognostic significances in the luminal A and TN subgroups but not in the HER2-positive subgroup, suggesting that the prognostic significance differs among the various breast cancer subgroups.

Proliferation has been recognized as a reliable breast cancer prognosticator [1], [2], [3], which fact is supported by global gene expression analyses showing the key biological drivers in prognostic signatures to be genes related to proliferation [17], [18], [19], or by the Oncotype DX multi-gene test, which contains five proliferation-related genes out of the 16 test genes [20]. Proliferation has been recognized also as a reliable predictor of the responses to adjuvant [21], [22], [23] or neoadjuvant chemotherapy [5], [10], [11], [24] in breast cancer. It seems, therefore, that measurement of proliferation activity has a great clinical potential as an effective tool for the management of breast cancer treatment. As the present study has confirmed the significant correlation of a new proliferation marker, the CPCC, with RFS, further studies on chromatin CKAP2 as a possible prognostic or predictive marker in the management of breast cancer are warranted.

For estimation of proliferation activity, the MAI has been studied extensively, and has shown consistent correlations with RFS in breast cancer [1]. Proliferation markers such as Ki-67 have been introduced to facilitate and standardize the estimation procedure, but there have been questions as to the prognostic significance of Ki-67 or any other such marker [3], [9], [21], [25], [26]; the guidelines of the American Society of Clinical Oncology, in fact, do not include Ki-67 on the list of routine prognostic tests [8]. Various methods by which proliferation activity in breast cancer is measured, showed conflicting results [27], and the controversies, might be related to the various phase-specificities of the proliferation markers: for example, while Ki-67 is present in all cell-cycle phases except G_0_, cyclin D and E show their activities only in the G1/S transition [6]. Therefore, further validation of the currently available proliferation markers, and development of additional markers, is required. In the present study, chromatin CKAP2, a new proliferation marker with its specificity in the mitotic phase, showed consistent RFS-correlation results across two institutions, with values higher than those for the MAI. As the use of IHC markers offers great potential advantages in regard to evaluation time and efficiency, CKAP2 immunohistochemistry could facilitate the clinical application of proliferation activity to breast cancer by providing a simple and effective MAI-alternative measure. However, as an international assessment of Ki67 has been convened [25], further study comparing chromatin CKAP2 with Ki-67 is warranted.

Although the prognostic significance of proliferation activity in breast cancer seems evident, the prognostic significance as measured by Ki-67 has been modest in breast cancer [8], [26]. Nonetheless, because breast cancer has been classified into distinct molecular subgroups based on receptor status [28], [29], the prognostic significances should be clarified in each subgroup, since any of them might be disclosed as having a clinically applicable potential. Recent retrospective studies suggest that proliferation activity is an effective prognostic marker only in the luminal A [21], [30] or TN [31] subgroup. Our results, showing prognostic significance in the luminal A and TN subgroups but not in the HER2-positive subgroup, are consistent in that regard. Our results additionally suggest that the prognostic significance of CPCC for group 2 differs between the luminal A and TN subgroups, though further independent validation is necessary. Further subgroup validation analyses could identify the best subgroup for clinical application of proliferation activity in breast cancer.

In summary, the present study has shown that chromatin CKAP2 is an effective independent prognostic marker for RFS in early-stage breast cancer. Further investigation of chromatin CKAP2’s clinical application to the management of breast cancer treatment, therefore, is warranted. Additionally, our results indicated that the prognostic significance of proliferation activity differs among the various breast cancer subgroups, which fact could potentially reveal the breast cancer subgroup wherein proliferation activity is important for survival prediction.
